# Complete Genome Sequence of *Pseudomonas viridiflava* CFBP 1590, Isolated from Diseased Cherry in France

**DOI:** 10.1128/genomeA.00662-17

**Published:** 2017-07-27

**Authors:** Michela Ruinelli, Jochen Blom, Joël F. Pothier

**Affiliations:** a Environmental Genomics and Systems Biology Research Group, Institute for Natural Resource Sciences, Zurich University of Applied Sciences (ZHAW), Wädenswil, Switzerland; b Bioinformatics and Systems Biology, Justus-Liebig University Giessen, Giessen, Germany

## Abstract

*Pseudomonas viridiflava* causes foliar and stem necrosis, as well as stem and root rot on a wide range of plants. We report here the first complete genome of a *P. viridiflava* strain, isolated from diseased tissue of a cherry tree.

## GENOME ANNOUNCEMENT

The pectinolytic bacterium *Pseudomonas viridiflava* is a multihost pathogen belonging to the *P. syringae* species complex. Phylogeny based on similarity of housekeeping genes placed the *P. viridiflava* species within phylogroup 7 (1, 2), which reflects the genomospecies 6 obtained by DNA-DNA hybridization (3). *P. viridiflava* was originally isolated from a dwarf bean plant in Switzerland and was then reported to be a natural pathogen of a wide range of plants (4), including tomato (5) and the model plant *Arabidopsis thaliana* (6). Recently, strains of *P. viridiflava* were isolated also from nonagricultural habitats (7), thus highlighting the adaptation potential of this species. Previous studies revealed the presence of two distinct phylogenetic clusters within the *P. viridiflava* species (8) and showed the presence of two mutually exclusive configurations of the type III secretion system (T3SS), namely, the tripartite pathogenicity island (T-PAI) and the single pathogenicity island (S-PAI), located in two different genomic locations (9). Recently, Bartoli et al. showed that the T3SS configuration was not linked to pathogenicity and that the only trait found to be correlated with pathogenicity was the presence/absence of the type III effector AvrE (10).

To date, a total of nine *P. viridiflava* whole-genome shotgun sequences are available in GenBank, but no complete genome sequence has been provided yet. In this study, the first complete genome of a member of the *P. viridiflava* species was obtained using PacBio single-molecule real-time (SMRT) read sequencing technology. The selected strain, *P. viridiflava* CFBP 1590, was isolated in 1974 in France from a sour cherry tree displaying symptoms of cortical necrosis.

Genomic DNA for PacBio whole-genome sequencing was extracted following the protocol described by Pitcher et al. (11). PacBio library preparation and sequencing were performed at the Functional Genomic Center Zurich (Zurich, Switzerland). SMRTbells were prepared using the DNA template prep kit version 2.0 (3 kb to 10 kb) (Pacific Biosciences, Menlo Park, CA, USA), and sequencing was performed on a PacBio RSII system (Pacific Biosciences) run with P4/C2 chemistry. Six SMRT cells yielded 464,727 reads, with an average length of 5,901 bp (for a total of 802,154,567 bp). The obtained reads were assembled into one single and circular contig of 6,035,297 bp using the HGAP3 approach on SMRT analysis software version 2.3.0 with manual refinement using BLASTn (12). The sequence was annotated using GenDB (13), which yielded a total of 5,129 genes and a GC content of 59.31%.

The obtained genome possesses the S-PAI T3SS configuration, as well as the AvrE T3E, found also in the draft genomes of *P. viridiflava* strains TA043 (GenBank accession number AVDV01000000), UASWS0038 (GenBank accession number AMQP01000000), and CC1582 (GenBank accession number AVDW01000000), isolated from cowslip, rhododendron, and epilithon, respectively (10). In contrast to the draft genome of *P. viridiflava* strain CDRTc14 (GenBank accession number MBPF01000000), no plasmid was found in the strain sequenced in this study.

### Accession number(s).

The complete genome sequence of *P. viridiflava* strain CFBP 1590 has been deposited in DDBJ/ENA/GenBank under the accession number LT855380.
